# Exploring prediction of tick and tick-borne encephalitis cases in Sweden using citizen science data

**DOI:** 10.1016/j.isci.2026.116879

**Published:** 2026-07-21

**Authors:** Yichao Liu, Junwen Guo, Peter Fransson, Stefan Widgren, Anna Omazic, Joacim Rocklöv

**Affiliations:** 1 Interdisciplinary Center for Scientific Computing, Heidelberg, Germany; 2 Department of Epidemiology and Global Health, Umeå University, Umeå, Sweden; 3 Heidelberg Institute of Global Health, Heidelberg University, Heidelberg, Germany; 4 Department of Epidemiology, Surveillance and Risk Assessment, Swedish Veterinary Agency (SVA), Uppsala, Sweden; 5 Department of Chemistry, Environment and Feed Hygiene, Swedish Veterinary Agency (SVA), Uppsala, Sweden

**Keywords:** Tick, TBE, citizen science, case counts mapping

## Abstract

Tick-borne encephalitis (TBE) remains a severe public health threat in affected areas with shifting geographic distribution linked to environmental change. However, systematic tick surveillance is limited due to the cost and effort of field monitoring. Here, we evaluate the potential of using national citizen science tick data reports for predicting tick-human interaction and TBE risk alongside socioeconomic and environmental data. We integrate citizen science observations with socioeconomic and environmental data, and apply statistical and machine learning models to identify key drivers and assess predictive performance. Among them, XGBoost achieved the highest accuracy for predicting tick report frequency and TBE cases. Rural population size, soil temperature, and vegetation index were key predictors of tick-human interaction, while tick report frequency, soil temperature, and diurnal temperature range were associated with TBE cases. These findings underscore the value of tick citizen science data for enhancing public health surveillance and prevention strategies.

## Highlights

• Rural population size, soil temperature and NDVI drive tick reports
• Soil temperature, DTR, and tick citizen science counts are key predictors of TBE
• XGBoost outperforms spatial LASSO and other tested models
• Citizen science data improves tick borne disease risk prediction

## Graphical abstract

**Figure:**
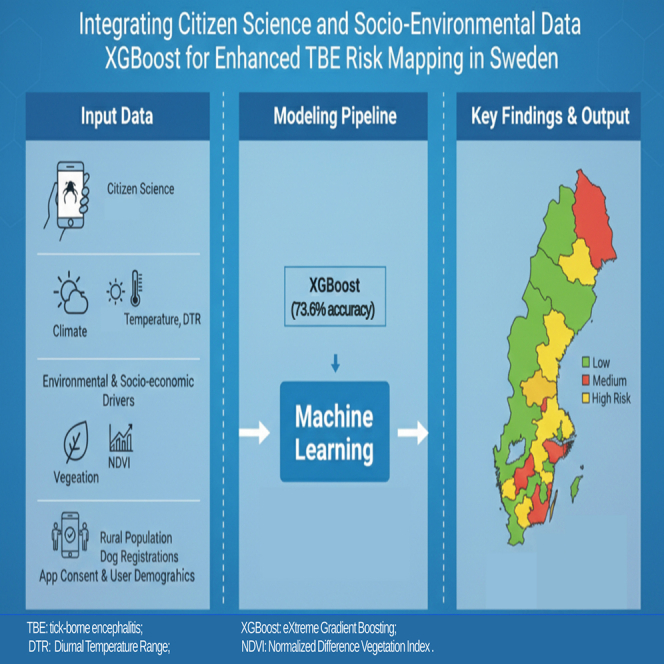

## Teaser

Health sciences; Disease; Tick; Biological sciences

## Introduction

Ticks serve as vectors for zoonotic infections, such as Lyme disease and tick-borne encephalitis (TBE), posing a significant public health risk in affected areas.^1^ Over the past decades, climate and land use changes have created more favorable conditions for tick survival and reproduction, and consequently lead to increased circulation and expansion of tick-borne pathogens and the incidence of tick-borne diseases.^2^^,^^3^ Locally, these changes often interact with other social and environmental conditions that determine the precise tick abundance and human disease incidence. The prevalence of *Ixodes ricinus* (a major vector for Lyme disease and TBE) is influenced by microclimatic conditions, including temperature, humidity, and precipitation. It is also affected by the environmental conditions, such as effects proxied by the normalized difference vegetation index (NDVI), reflecting the density and greenness of vegetation,^4^ and habitat richness, determining reservoir host abundance, predation and pathogen dilution, proxied by the normalized habitat richness (NHR).^4^ Research has shown that for Europe, temperatures above 7°C–8°C, a relative humidity exceeding 70%–80%, and moderate soil moisture favor tick survival.^5^^,^^6^ Consequently, rising temperatures and milder winters have extended tick activity periods, and enabled them to thrive in previously unsuitable regions.^7^^,^^8^^,^^9^ A recent study suggested that TBE risk decreases in highly diverse habitats, indicating that high NHR may be associated with lower tick survival.^10^ Changes in land use, such as reforestation and urban expansion, create new habitats for ticks to proliferate and may result in increased human exposure risks.^11^ In addition, land use patterns and population density may also shape tick habitats by influencing host availability and tick-human encounter rates. These influences are particularly evident for pets and grazing animals, which are vulnerable to tick bites and thus, are important subjects for tick surveillance efforts.^12^^,^^13^ Collectively, these environmental and socioeconomic drivers shape the distribution and abundance of tick populations and circulation of tick-borne pathogens, such as TBE virus (TBEV). In Sweden, tick populations are dominated by two Ixodes species: *Ixodes ricinus*, which is widespread throughout most of the country,^14^ and *Ixodes persulcatus*, which only occurs in the northern regions.^15^ Among them, *I. ricinus* is the most common tick species and a primary vector of several tick-borne diseases, including Lyme borreliosis and TBE.^15^

To address the rising tick-related public health challenges, it has become more important to predict changes in tick-human interaction and circulation of TBEV, and a frequent approach taken has been the deployment of niche modeling or species distribution modeling (SDM).^16^^,^^17^^,^^18^^,^^19^ These models integrate ecological, climatic, and land-use variables to estimate the potential range and abundance of tick populations or human disease risk. Importantly, understanding tick-human interaction provides a foundation for predicting the risk of tick-borne infections such as TBE. Common SDM approaches are used for tick and TBE prediction include: maximum entropy (MaxEnt),^20^ generalized linear models (GLMs), generalized additive model (GAM),^21^ least absolute shrinkage and selection operator (LASSO) regression,^22^ and ensemble modeling techniques.^19^ These have been successfully applied to predict the spread of ticks in response to climate change.^23^^,^^24^^,^^25^ The accuracy of SDMs depends on the availability of valid presence and absence data. Presence-only models, such as MaxEnt, rely on observed occurrences and background data. In contrast, models such as GLM and GAM force the inclusion of often unvalidated pseudo-absences data alongside presences.^26^

Although these models each have their strengths and weaknesses, their effectiveness often largely depends on the quality of surveillance data used to develop and evaluate the models, and a critical gap in data concerns the availability of local occurrence and abundance data of different tick species. The data gap is reflected in the labor intensive effort of systematic tick surveillance collections.^27^^,^^28^^,^^29^^,^^30^ Active surveillance involves drag sampling techniques, which, while effective, require labor-intensive manual work, making nationwide implementation impractical. In contrast, passive surveillance relies on routine reports from the healthcare or veterinary providers, which are often delayed and primarily limited to populated areas, creating gaps in data coverage. As a more scalable and affordable complement, citizen science approaches to tick surveillance have emerged in recent years. Its passive data streams offer a cost-effective and scalable means of monitoring tick-human interaction and the associated risk of tick-borne infections.^31^^,^^32^^,^^33^ Importantly, these reports do not represent systematic measurements of tick abundance; rather, they arise from a joint process driven by tick presence and human exposure behavior. Existing studies primarily used citizen science data for surveillance or short-term prediction.^34^^,^^35^ However, none of them integrate such data into a disease modeling framework for TBE. Consequently, engaging the public in tick surveillance efforts can provide extensive data on where and when humans encounter ticks that may indirectly inform tick ecology, tick seasonal activity patterns, their occurrence in new environments, and perhaps include valuable information for understanding the risk of vector-borne disease better. Tick citizen science data can, therefore, fill the data gaps and support early detection of tick emergence and associated disease risks. However, despite its potential, citizen science data also has limitations: it is often spatially and temporally uneven due to the unsystematic context-dependent sampling. To date, it has rarely been used for tick risk mapping or tick-borne disease risk prediction, and its usefulness remains to be tested.

**Figure 1:**
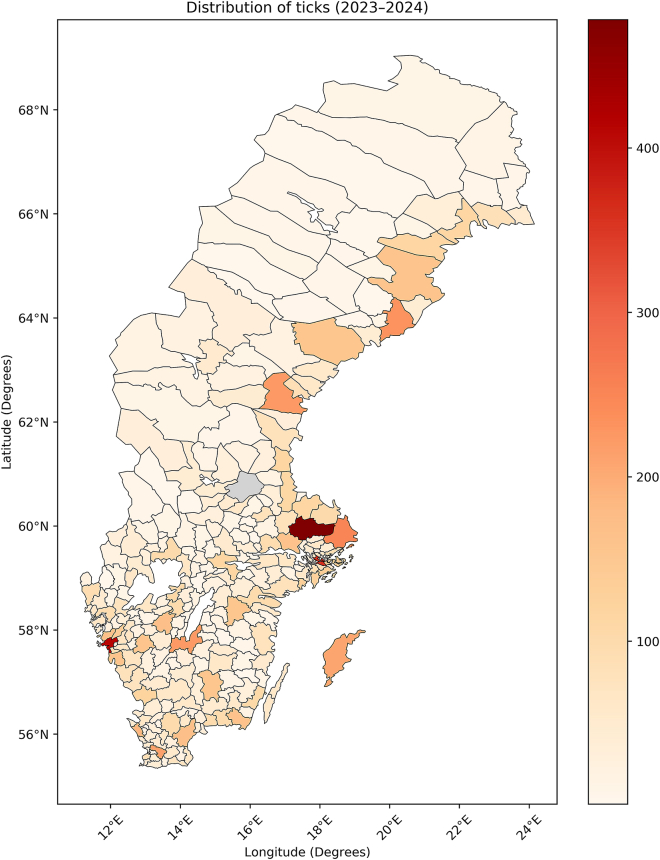
Annual citizen science tick reports in 2023–2024 at a municipality level in Sweden Color intensity represents the total number of reported ticks. Gray areas are the municipalities that do not have any reported ticks from citizen science.

In this study, we addressed two objectives: first, we model citizen science tick data, citizen-reported *Ixodes* spp. tick observations submitted through the Swedish Veterinary Agency (SVA), and test what factors best predict the tick-human interaction. Different models were tested and evaluated. The tick report frequency data were collected via a web-based tool Rapportera Fästing,^36^ where users submitted tick images and related information following instructions, such as location and host, provided on the website. Building on the tick modeling results, we model municipal-level TBE case counts (Figure 1) in two separate ways to assess whether the citizen science data are a significant predictor of TBE risk. Different models were tested and evaluated for this purpose and, specifically, TBE cases were modeled (1) using observed tick report frequency and (2) using model-predicted tick report frequency, each considered independently alongside established social and ecological covariates. We aggregated the data spatially at the municipality level, and temporally for the years 2023 and 2024. Overall, this study illustrates how integrating environmental, climatic, and socioeconomic data with citizen science tick reports can support an integrated one health surveillance system and provide added value for predicting and monitoring TBE risk.

## Results

### Best prediction performance

**Figure 2:**
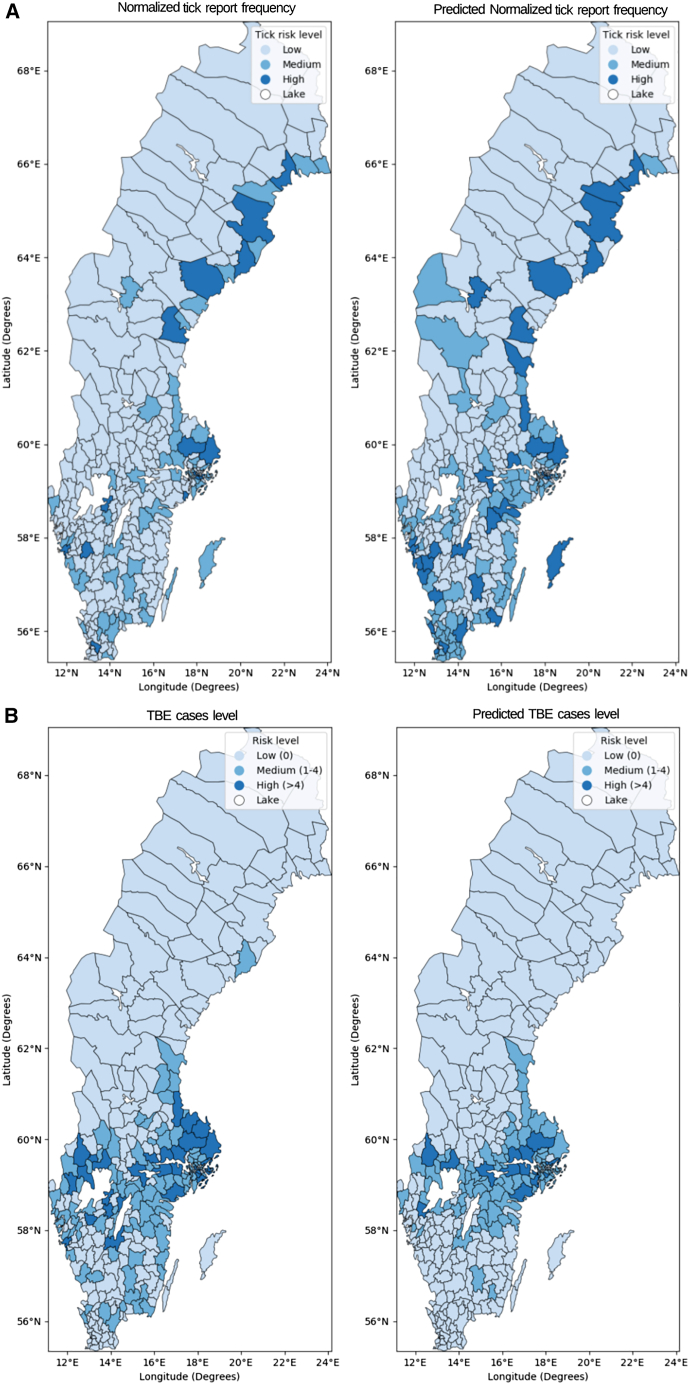
Geospatial frequency map for normalized tick report and TBE (A) The left map is the citizen science ground-truth-normalized tick report frequency map, where different levels are categorized by Gaussian mixture model (GMM) method. The right map is the prediction by extreme gradient boosting (XGBoost), where predicted levels are categorized by GMM method. (B) The left map is the ground truth tick-borne encephalitis (TBE) case level map, whose different levels are clustered by the GMM method. The right map is the prediction of TBE case level by XGBoost, whose predicted levels are clustered by the GMM method. Low represents no TBE cases. Medium represents 1–4 TBE cases. High represents higher than 4 cases.

We find that the extreme gradient boosting (XGBoost) model is slightly better than other models in predicting areas with normalized tick report frequency (Table 1). The corresponding performance of XGBoost for prediction of normalized tick report frequency is illustrated in Figure 2A. From Figure S1 in supplemental information, we can see that the model cannot consistently distinguish medium tick report frequency level from high and low tick report frequency level. In spatial terms, the model underestimates tick report frequency in western Sweden and overestimates some municipalities in southern Sweden (Figure 2A). From the map, it is evident that the most tick activity was reported in coastal regions, with southern areas generally reporting more ticks than northern regions. The covariate selection process for all models and hyperparameters are shown in the supplemental information (Tables S2–S8).

**Table 1:** Model comparison on the 3-class categories for tick and TBE data


	GLM (base covariate set)	GLM (selected model)	GAM	LASSO regression	Spatial LASSO regression	Decision tree	XGBoost
**Tick report frequency model comparison**							

Accuracy	37.17%	65.28%	60.63%	63.57%	66.91%	67.29%	68.77%

**TBE model comparison with normalized tick report frequency**							

Accuracy	60.22%	61.34%	68.03%	63.94%	63.94%	67.66%	73.61%

**TBE model comparison with estimated normalized tick report frequency**							

Accuracy	47.12%	49.54%	60.59%	62.08%	62.08%	60.62%	69.89%

The reported accuracy was computed on the 2024 test set. Categories were created by using the Gaussian mixture model (GMM) clustering method. Abbreviations: GLM, generalized linear model; GAM, generalized additive model; XGBoost, extreme gradient boosting; LASSO: least absolute shrinkage and selection operator.

The performance of the selected model for TBE cases level prediction is presented in Table 1, while the covariate and hyperparameter selection are provided in the supplemental information (Tables S9–S14). Overall, models incorporating citizen science tick reports outperform those using the predicted normalized tick report frequency from the model described earlier. In addition, more complex models generally exhibit smaller performance degradation than simpler models. Among all models, XGBoost achieved the best performance, significantly outperforming other methods. The corresponding ground-truth-predicted TBE cases map on the basis of the categories established with GMM and confusion matrix are shown in Figure 2B and in the supplemental information (Figure S2). In these figures areas classified as high cases along the east coast are predicted as medium cases, while several medium cases regions in the south are predicted as low cases. In addition, the confusion matrix, the ground truth, and the predicted TBE case map based on the categories clustered by K-means method are shown in Figures S3 and S4. The accuracy of XGBoost on the cluster of GMM method is 73.61%, while on the cluster of K-means method is 90.33%. From the model selection for the XGBoost model, we found that precipitation in warm season and total land use are additional covariates that contribute to model performance (Table S14). Therefore, the final selection of covariates includes, in order: base covariate set, precipitation warm (precipitation in warm season), and the total land use.

### The most important predictors of tick and TBE cases

**Figure 3:**
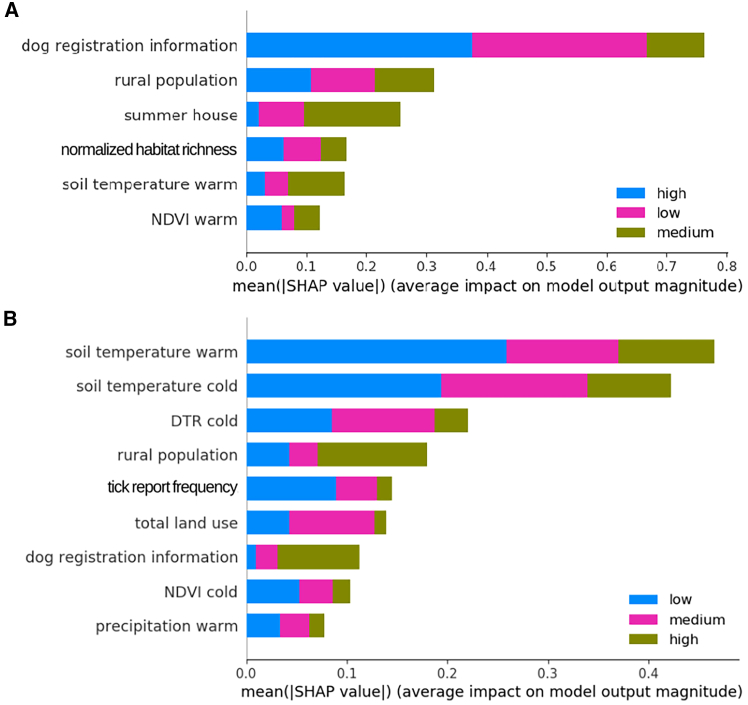
Calculated SHAP value of the importance of predictors for the different selected XGBoost models (A) Tick report frequency level and (B) tick-borne encephalitis (TBE) case level separated into categories of low, medium, and high observations. The rank is based on the total contributions, and the length of different colored bars shows the contribution to each level. Note: DTR, diurnal temperature range; NDVI, normalized difference vegetation index.

Shapley additive explanations (SHAP) and GLM covariates analyses were conducted specifically for citizen science tick data to understand the contribution of the covariates,^37^^,^^38^ with results detailed in Figure 3A and Table S15, respectively. A discrepancy was observed between these two methods: while the GLM results highlight the significant influence of warm season soil temperature and NDVI, the SHAP analysis suggests that dog registration information exerts a disproportionately high influence, especially within low and high cases categories. Notably, the rural population remained a consistent predictor across both models, underscoring its central importance in understanding tick distribution.

In the TBE case mapping, the contribution of each feature to different case levels, calculated via SHAP values, is illustrated in Figure 3B. These results largely mirror the patterns identified in the GLM covariate analysis (Table S16). Interestingly, although NDVI during the cold season was statistically significant in the GLM covariate analysis, neither it nor tick report frequency emerged as the highest contributors across the three case levels in the SHAP analysis. Nevertheless, tick report frequency remains a notable predictor, ranking as the third most influential variable for identifying low TBE cases and consistently outperforming cold season NDVI.

## Discussion

In this work, we develop data-driven models to predict the geographical distribution of tick report frequency measured by citizen science reports and TBE case counts across Sweden. We identify the key climatic and socioeconomic covariates driving the patterns. Models selected and incorporated tick citizen science data, which proved valuable for TBE case level prediction. The tick report frequency citizen science data were the second most important feature after temperature (Table S15), validating our hypothesis that citizen-reported observations serve as a good proxy for tick-human interaction and serve as a potential proxy for TBE cases.

Our study differs from that of Ma et al.,^39^ who observed a region-scale decline in TBE cases despite climatic conditions that typically favor tick expansion. Ma el al. attributed this negative relationship to factors such as increased vaccination coverage in endemic areas, improved public awareness, behavioral changes that reduce exposure to ticks, and the expansion of surveillance and prevention programs.^39^ Our analysis focuses on spatial differences, which means that the connection we see between tick reports and TBE cases corresponds to differences across locations rather than temporal changes. The observed positive association likely arises because both citizen science tick observations and reported TBE cases are influenced by human presence and activity. In other words, although citizen science data are biased toward areas with more populations, this bias aligns with the spatial pattern of actual TBE cases, making tick report frequency a useful predictor. Consistent with this, a previous study shows that while tick abundance alone is not the primary driver of TBE human case counts, it is a necessary foundation for its focal distribution. This indicates that tick abundance data are necessary but not sufficient for precise TBE cases mapping.^40^ Tick report counts in both the GLM and SHAP analyses exhibit a consistent pattern, demonstrating that while they are significant predictors, they do not carry the same weight as temperature-related factors such as soil temperature and diurnal temperature range (DTR). This likely reflects the hierarchical nature of ecological drivers; climatic variables establish the fundamental physiological limits for tick survival and development, whereas tick report frequency represents a discrete observational realization of these underlying conditions. However, citizen science tick reports remain a critical indicator for public health. While the influence of environmental drivers such as temperature partly go beyond human control defining the tick habitat, within these suitable thermal ranges, citizen science tick data provide the necessary spatial resolution to differentiate between low and high case areas. Overall, tick report frequency index remains significant in both GLM coefficients and SHAP values, underscoring their potential role in TBE surveillance.

As ticks spend much of their life in the litter and upper soil layers, soil temperature is a critical factor in tick survival. Larval and nymphal stages are especially sensitive to low soil temperature, which leads to slow development and delayed questing, while excessively high summer soil temperatures can increase desiccation mortality.^41^ The study also shows that integrating soil temperature into predictive models allows better capture of microhabitats where ticks persist despite broader climatic constraints.^41^ The same results are shown in TBE cases modeling in Table S16 and Figure 3B. The SHAP analysis provides a clear quantification of the relative importance of environmental and demographic covariates in shaping TBE case level (Figure 3B). Soil temperature plays the most important role among all the covariates, which highlights the dual ecological importance of soil temperature, warm conditions promote tick activity and host-seeking, while cold periods regulate seasonal cycles and may synchronize host-vector interactions, both ultimately shaping TBE cases.^42^^,^^43^

Medium tick report frequency is often predicted as low tick report frequency or high tick report frequency level, which is likely due to the medium class not being well separated under the three-class clustering (supplemental information, Figure S2). As discussed earlier, citizen science reports were much higher in 2023 compared with 2024. We expect that if the citizen science project continues, the data will become more balanced across different years. Thus, we can avoid normalizing the tick report frequency. Figure 2A illustrates the geographical distribution of tick prediction errors, showing that many southeastern coastal municipalities are predicted incorrectly. This may be due to the model’s limited ability to distinguish low and medium tick report frequency.

Our predictions based on GMM categories (Figure S1) indicate that both low case and medium cases areas are frequently misclassified, suggesting that the model struggles to accurately predict the presence of TBE cases. We also tested a quantile-based approach, dividing tick report frequency into three equal-sized categories, but this method performed substantially worse than the unsupervised clustering approach. In addition to using observed tick report frequency, we also incorporated model-estimated tick report frequency as an alternative predictor for TBE cases. The results show 69.89% for GMM categories, which was lower than that attained by using the crude unprocessed tick reporting data (Table 1). A further observation was that several municipalities in the north-eastern coastal and western regions are predicted incorrectly (Figure 2B). Most of the south-western areas are true medium case areas but are predicted as low case areas. However, the number of citizen science reports from many of those municipalities in both 2023 and 2024 are relatively low. We believe this may be due to lower participation in the citizen science project in many of these regions compared with others, which results in fewer reported ticks and consequently leads the model to underestimate the true TBE cases. In contrast, the eastern coastal regions are often predicted as high case area but reclassified to medium case area, likely due to class imbalance between high and medium case levels.

In this work, we demonstrate that citizen science data can be leveraged to predict TBE case distributions across Sweden. We also investigate the meteorological and socioeconomic covariates that contribute to the prediction of tick report frequency and how it interplays with citizen-reported tick data to predict TBE. Comparing among a range of different modeling strategies, the most predictive model was XGBoost. The most influential variables were soil temperature and rural population for ticks and for TBE case counts. With citizen science data being an important indicator for TBE case counts, future work should further investigate its potential in early warning systems for tick-borne disease risk.

### Limitations of the study

There are several limitations of using citizen science data for TBE and tick modeling. Although our study shows a strong connection between citizen science tick data and TBE cases, the limited TBE cases data and citizen science tick data constrained the overall predictive power and generalization power of our model. Nonetheless, our findings indicate that citizen science observations have clear potential to enrich One Health surveillance frameworks by expanding spatial coverage and complementing traditional monitoring. Further on, future research should focus on leveraging longitudinal citizen science datasets, alongside small mammal surveillance data, to evaluate the potential of early tick population indicators to forecast TBE cases to follow in the seasons, enhancing proactive disease surveillance and prevention.^35^ The most prominent issue of concern with using citizen science data relates to the consistency of public participation. Citizen involvement is often high at the start of an initiative but tends to decline over time, resulting in temporal bias in reporting frequency. Such fluctuations in participation can distort temporal trends in tick activity and lead to biased model estimates.This limitation restricts the applicability of the GLM and GAM models. To avoid relying on the 2024 maximum tick report frequency and to balance tick report frequency between 2023 and 2024, we normalized the tick report frequency for both years and used them as a proxy for tick report frequency. However, the current GLM and GAM implementations only support continuous distributions such as Gaussian or Gamma, which are not appropriate for normalized tick report frequency data. As a result, we were required to include the 2024 maximum tick report frequency as an offset term, information that would not be available in a real-world predictive setting, as shown in Table S18. In addition, reporting effort is rarely standardized or quantifiable, making it difficult to distinguish true absences from cases where no reports were submitted. Consequently, absence data cannot be reliably interpreted as true biological absences, limiting the applicability of certain statistical modeling approaches and complicating inferences about temporal changes in tick abundance or distribution patterns.

## Resource availability

### Lead contact

Requests for further information and resources should be directed and will be fulfilled by the lead contact, Joacim Rocklöv (joacim.rocklov@umu.se).

### Materials availability

This study did not generate new unique materials.

### Data and code availability

• All data reported in this publication will be shared by the lead contact upon request.
• Code used in the analysis will be made publicly available immediately after publication through this link https://github.com/Christian-lyc/citizen_science_tick_analysis.git.
• Any additional information required to reanalyze the data reported in this article is available from the lead contact upon request.

## Acknowledgments

Y.L., P.F., and J.R. were supported by the 10.13039/100005156Alexander von Humboldt Foundation. We acknowledge that the present contribution is supported by the 10.13039/501100009318Helmholtz Association under the joint research school HIDSS4Health Helmholtz Information and Data Science School for Health. Overall, the study was partly supported by IDAlert, which has received funding from the European Union's Horizon Europe programme under grant agreement no. 101057554. We are grateful to the Public Health Agency of Sweden for providing the TBE case data used in this study.

## Author contributions

Conceptualization, Y.L., P.F., J.G. and J.R.; methodology, Y.L., P.F. and J.G.; software, Y.L.; validation, J.G., P.F., A.O., S.W. and J.R.; formal analysis, Y.L., investigation, P.F., J.G. and J.R.; writing – original draft, Y.L.; writing – review and editing, P.F., J.G., A.O., S.W., and J.R.; funding acquisition, J.R.; supervision, P.F., J.G. and J.R.

## Declaration of interests

The authors declare no competing interests.

## STAR★Methods

### Key resources table

**Table:** 

REAGENT or RESOURCE	SOURCE	IDENTIFIER
**Software and algorithms**		

Python (v3.11.2)	Python Software Foundation	https://www.python.org/
Xgboost	Chen and Guestrin, 2016	https://xgboost.ai/; RRID: SCR_021361
pyGAM library	Daniel Servitje	https://github.com/dswah/pyGAM; RRID: SCR_025211
statsmodels python package	Seabold and Perktold, 2010	https://www.statsmodels.org/; RRID: SCR_016074
Citizen Science Tick Analysis Code	This paper	GitHub: https://github.com/Christian-lyc/citizen_science_tick_analysis

**Deposited data**		

TBE notification records	Swedish Public Health Agency	https://www.folkhalsomyndigheten.se/
Citizen science tick reports	Swedish Veterinary Agency (SVA)	https://rapporterafasting.sva.se/introduction
ERA5-Land climate reanalysis	Copernicus Climate Change Service	https://cds.climate.copernicus.eu/
MOD13Q1.061 NDVI	NASA LP DAAC	https://earthengine.google.com/
Socioeconomic statistics	Statistics Sweden (SCB)	https://www.scb.se/en/finding-statistics/

### Experimental model and study participant details

#### Sample description

The citizen science participants data is collected by SVA from May, 2023 to December 2024 at municipal level in Sweden. No personal information involved in the submission report.

#### Ethics statement

The data used in this study was secondary data and deidentified so no ethical approval was needed to conduct this study.

### Method details

#### Data on tick-borne encephalitis

We obtained data on TBE from the Swedish public health agency. The dataset covers calendar years 2023 and 2024. Case records are aggregated individual notifications to the municipal level for consistency with environmental and demographic covariates.Case counts are attributed to the reported location of infection. Across the two-year period (2023–2024), the agency recorded a cumulative total of 883 confirmed TBE cases.

#### Tick citizen science data

A tick citizen science tool was launched in May 2023 by the Swedish Veterinary Agency and since then collections have continued. The tool allows citizens to share photos of ticks they encounter (i.e., from themselves, pets or in the environment) along with information of time, location if the tick was found in the environment or on a host to the project website. Most reports are submitted by dog and cat owners, which means the dataset is heavily influenced by pet-associated ticks rather than ticks from wildlife. Entomologists at SVA then classify the reported tick photos to genus level. In this study, we used the tick data from May 2023 to the end of 2024. Approximately 99.9% of the reported ticks are identified as *Ixodes* spp., so species from other genera are excluded from the analysis. Tick reports are aggregated at the annual and municipal levels. The aggregated data is presented in Figure 1. From the figure, it is evident that, despite only having eight months of data in 2023, the highest tick report frequency is still higher than those in 2024. This is likely due to the initial surge of public interest and more enthusiastic participation at the beginning of the citizen science initiative, leading to a bias compared to 2024. To reduce this bias and balance the data distribution between the two years, we excluded the first month, May 2023, from the analysis.

#### Climate and environmental data

We obtained climate data, including 2-meter air temperature, soil temperature, precipitation, minimum and maximum temperature, and dew point temperature, from the ERA5-Land daily aggregated climate reanalysis dataset,^44^ and Normalized Difference Vegetation Index (NDVI) from the MOD13Q1.061 Terra Vegetation Indices^45^ via Google Earth Engine. The data were aggregated at the monthly and municipal levels to align with the tick data. We calculated several covariates similar to bioclimatic variables. These are climate-derived indicators that describe long-term environmental conditions relevant to species survival and distribution. Examples include the annual temperature range (the difference between maximum and minimum temperatures), the annual mean temperature, and the annual precipitation. As described in the tick data section, we model climate data spatially rather than temporally. Additional covariates, including Diurnal Temperature Range (DTR, calculated similarly to the temperature range), precipitation, soil temperature, 2-meter air temperature, NDVI, and humidity, were also aggregated separately for the warm season (June to August) and the cold season (December to February). Humidity was estimated from dew point temperature using the formula in paper.^46^ In addition, Dagostin et al. have shown that high habitat richness is associated with a reduced risk of TBE.^10^ Following this study, an NHR index was calculated and included as an additional environmental covariate (more details about calculation, such as habitat richness dataset, can be found in the Cervellini et al. study^4^).

#### Socioeconomic data

Since the tick citizen science data reflects human–tick interactions, socioeconomic information is important for accurate prediction. We collected data on dog registrations, urban and rural population distribution, recreational land use, total land use, the number of summer houses (as an indicator of seasonal human presence in nature), and the Gini index measuring income or wealth inequality among the population, from Sweden statistics database.^47^

#### Covariates selection

To reduce multicollinearity and avoid autocorrelation among features, we calculated the Variance Inflation Factor (VIF) and excluded covariates with VIF values greater than ten.^48^ Specifically, we removed cold season temperature, warm season temperature, urban population, annual temperature, annual precipitation, and temperature range. These exclusions were based on the following correlations: cold and warm season temperatures are highly correlated with soil temperature, annual temperature, and temperature range; urban population is correlated with dog registration data; and annual precipitation is correlated with both warm and cold season precipitation. The full set of covariates used in the modeling is shown in Table S1. Additionally, we assumed a latency between climate variables and tick reports,^49^ using climate conditions from 2022 as predictors for the normalized tick report frequency in 2023. The same lag was applied between tick reports and TBE case data as well.

#### Modeling approaches

We integrated all data to municipality level, including tick, TBE, climate data, environmental data and socioeconomic data. Using this integrated dataset, we applied five modeling approaches for normalized tick report frequency and TBE cases mapping, followed by two clustering methods for comparative analysis. We modeled normalized tick report frequency and TBE cases for the year 2024 to assess the contribution of individual covariates. A full list of considered covariates is shown in Table S1. To achieve this, we first applied a GLM to the candidate drivers, to identify the key covariates and establish a baseline model. We then performed model selection for both normalized tick report frequency and TBE cases modelling. In total, we performed model selection and evaluation for five models: GLM, GAM, LASSO, decision tree, XGBoost, along with an additional spatial LASSO model. The modeling approaches are briefly described below and the detailed model comparison part is in next section:

GLM^21^: GLM is a flexible extension of ordinary linear regression that allows response variables to follow distributions other than the normal distribution. It links the expected value of the response variable to a linear combination of predictors through a link function, enabling the modeling of binary, count, and continuous outcomes within a unified framework.

GAM^21^: GAM extends the Generalized Linear Model by allowing nonlinear relationships between predictors and the response variable through smooth functions. Instead of assuming a strictly linear effect, GAMs model the expected response as the sum of smooth functions of the predictors, linked to the mean via a suitable link function.

LASSO^22^: LASSO is a regularization technique for regression models that simultaneously performs variable selection and coefficient shrinkage. By adding an L1 penalty term to the loss function, LASSO constrains the sum of the absolute values of the regression coefficients, forcing some of them to become exactly zero.

Decision Tree^50^: Decision tree is a nonparametric supervised learning method used for classification and regression tasks. It models decisions by recursively splitting the data into subsets based on feature values, forming a tree-like structure of decision rules. Each internal node represents a test on a predictor, each branch a possible outcome, and each leaf node a predicted class or value. Here we use the regression model.

XGBoost^51^: XGBoost is an optimized implementation of the gradient boosting framework designed for speed, scalability, and performance. It builds an ensemble of decision trees sequentially, where each new tree corrects the errors of the previous ones by minimizing a differentiable loss function.

GMM^52^: Gaussian Mixture Model is a probabilistic model that represents data as a weighted sum of multiple Gaussian distributions. GMMs are widely used for clustering.

K-means^53^: K-means is a simple, non-probabilistic clustering algorithm that partitions data into K clusters by minimizing within-cluster variance.

#### Normalized tick report frequency mapping

We investigate the influence of covariates on the tick report frequency. We use climate and environmental factors as well as socioeconomic factors for each municipality to predict normalized tick report frequency. We first perform covariate analysis with a GLM model (binomial distribution with log link function) for tick report frequency and find the base covariate set to build the baseline models from. The negative binomial distribution was chosen over the Poisson distribution, due to overdispersion in the data. Based on this base covariates set, to model normalized tick report frequency, we conducted model selection for each model approach and a final cross-model-approach comparison of the selected models, including both classification and regression approaches. We have chosen Gaussian Mixture Model (GMM)^52^ and K-means^53^ to cluster the tick report frequency data into three categories and binary categories. Population size was included as offset terms in both the GLM and GAM models. The GLM formulation for tick modeling, utilizing min-max normalization to scale the intensity to a range of [0,1], is shown in Equation 1.

$$Y_{i}∼negativebinomial\left(\mu_{i}\right)$$

(Equation 1) $$\log\left(\mu_{i}\right)=X_{i}^{T}\beta +\log\left(pop\right)+\log\left(\max\left(Y\right)\right)$$

Where *Y* denotes the tick report frequency and *X* represents the covariates. To account for the higher expected frequency of tick reports in densely populated areas, population size was included as an offset term. This ensures the model reflects relative tick abundance rather than human population density. To mitigate differences in tick report frequency magnitude between 2023 and 2024, the annual maximum tick report frequency was also included as an offset. By doing so, we assume that people’s interests are focused on the relative normalized tick report frequency levels within a given year, rather than on comparisons across different years. In the model selection and comparison, the 2023 tick citizen science data were used for training, while the 2024 data served as the test set. Spatial 5-block cross-validation was applied to the training data. For regression models, the predicted normalized tick report frequency were mapped into corresponding count levels based on these clusters, while for classification models, the clustered count levels were directly used for model fitting. The set of covariates for the final selected model was determined through an add-on feature selection process starting from the base covariates set. Additional covariates were then added stepwise, one at a time, by adding the covariate which led to the largest reduction in cross-validation error at a specific step. This process was repeated until no further performance improvement was observed, i.e., when none of the remaining covariates could improve cross-validation performance. The same model selection procedure is performed for all model categories. Since we are modeling on spatial data, a naive idea is that neighboring municipalities should have similar prediction errors. Motivated by prior work on graph regularization,^54^ we introduce a spatial error term to regularize the LASSO regression model by imposing a penalty on discrepancies in prediction errors between neighboring regions, namely spatial LASSO regression. The details can be found in Methods S1.

#### TBE cases modeling

In the same way as for tick report frequency modeling, we applied a GLM with a negative binomial distribution to the TBE case data. This model was used to analyze the climate, environmental and socioeconomic covariates, along with the normalized tick report frequency data, to identify a set of significant base sets of covariates. The 2023 TBE data were used for training, while the 2024 data served as the test set. Here, we use both real tick citizen science data and the predicted normalized tick report frequency from tick modeling. After model selection, all models are compared based on model performance. The best performing model was then trained on the 2023 dataset and evaluated using the 2024 data.

### Quantification and statistical analysis

#### Software and approach

All data curation and preprocessing workflows were implemented in Python v.3.11.2. Specifically, pandas and NumPy were utilized for data frame manipulation and cleaning, while pygam and XGBoost libraries facilitated the preliminary analysis and tabular organization required for the distribution modeling.

#### Statistical method

We used GMM and K-means for tick citizen science data and TBE cases clustering. Negative binomial GLM, Poisson GAM, LASSO regression, Decision tree and XGBoost were used for TBE and tick modeling. Statistical significance of individual covariates in the negative binomial GLM was assessed using two-sided Wald z-tests, as implemented in the statsmodels package; covariates with *p* < 0.05 are marked with asterisks in Tables S15 and S16.
