# Supplementary material for: Exploring prediction of tick and tick-borne encephalitis cases in Sweden using citizen science data
Source: iScience. 2026 Jul 21;29(8):116879. doi: 10.1016/j.isci.2026.116879 (PMC13393814; doi:10.1016/j.isci.2026.116879)
Supplement: Document S1. Figures S1–S5, Tables S1–S18, and Methods S1 [file mmc1.pdf]

iScience, Volume *29*

Supplemental information

Exploring prediction of tick and tick-borne encephalitis cases in Sweden using citizen science data

Yichao Liu, Junwen Guo, Peter Fransson, Stefan Widgren, Anna Omazic, and Joacim Rocklöv

Supplemental information

Supplementary Figures

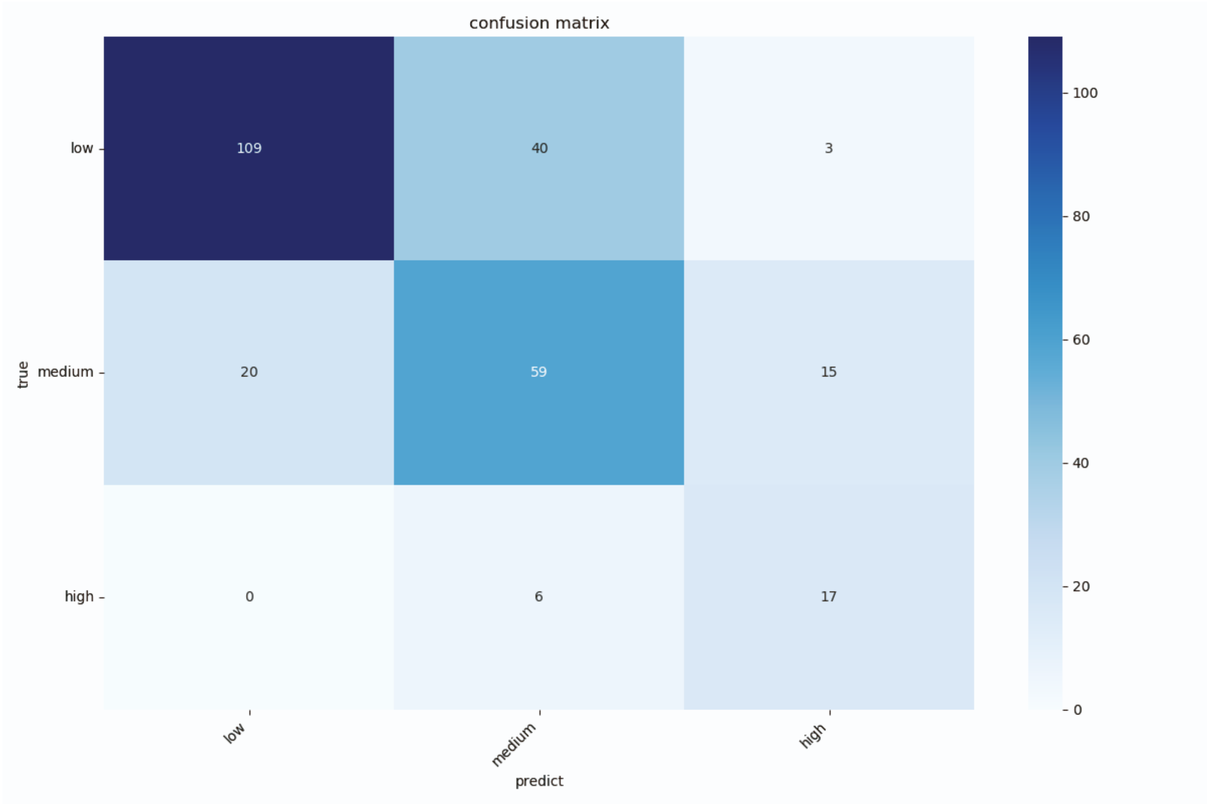

Fig. S1 Confusion matrix of eXtreme Gradient Boosting(XGBoost) for normalized tick report frequency prediction based on Gaussian Mixture Model(GMM) clustering method.

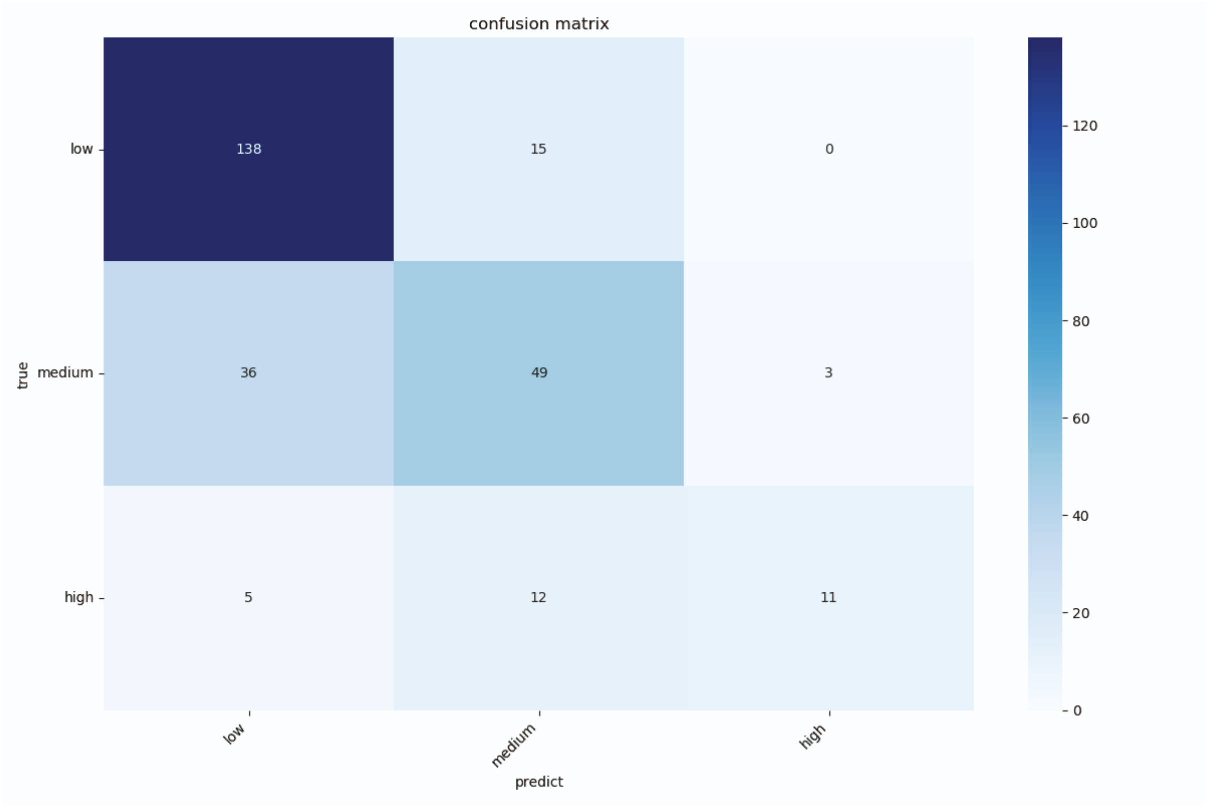

Fig. S2 Confusion matrix of eXtreme Gradient Boosting(XGBoost) for Tick-Borne Encephalitis(TBE) cases prediction based on Gaussian Mixture Model(GMM) clustering method.

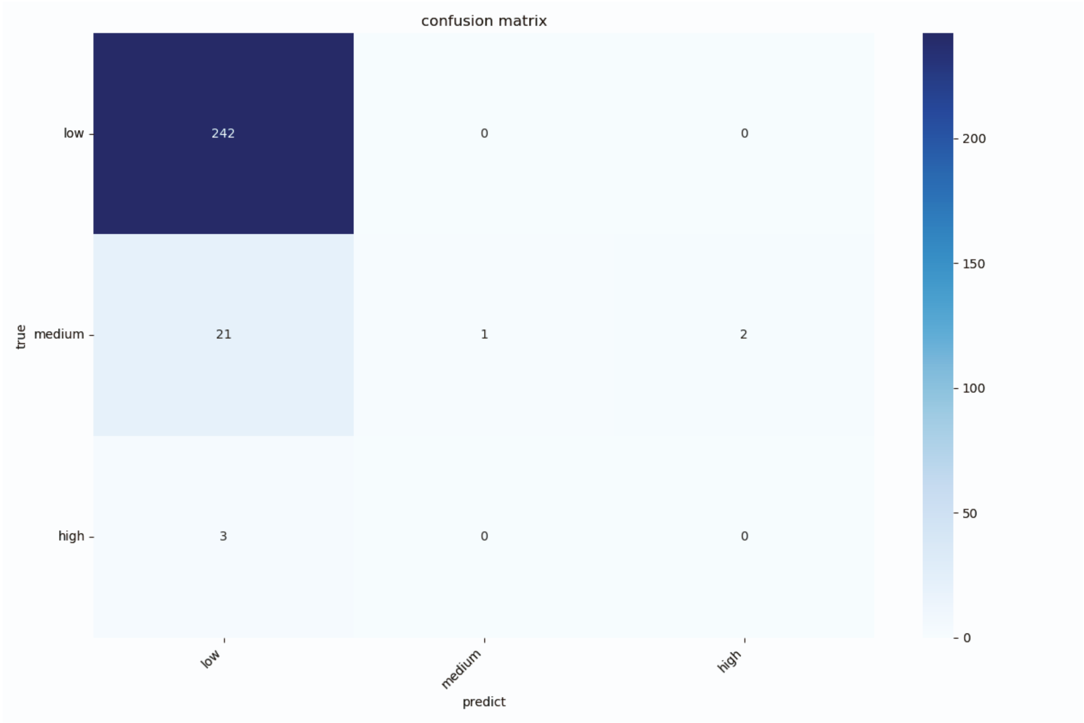

Fig. S3 Confusion matrix of eXtreme Gradient Boosting(XGBoost) for Tick-Borne Encephalitis(TBE) cases prediction based on K-Means clustering method.

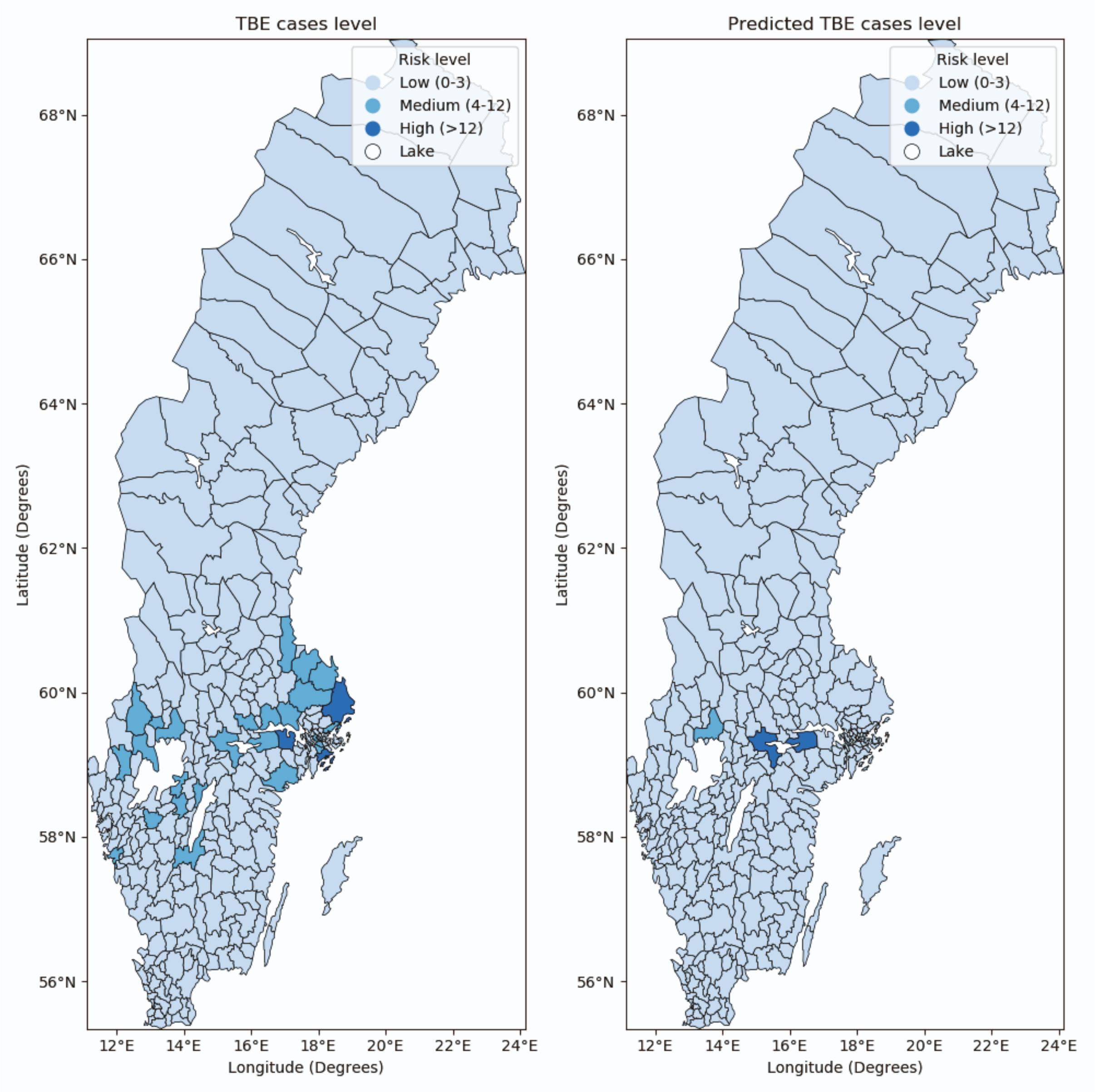

Fig. S4 Tick-Borne Encephalitis(TBE) cases map predicted by eXtreme Gradient Boosting(XGBoost) based on K-Means clustering method. The left map is the ground truth TBE cases map, whose different levels are clustered by K-Means method. The right map is the prediction of XGBoost, whose predicted levels are clustered by K-Means method. Low represents 0-3 TBE cases. Medium represents 4-11 TBE cases. High represents higher than 12.

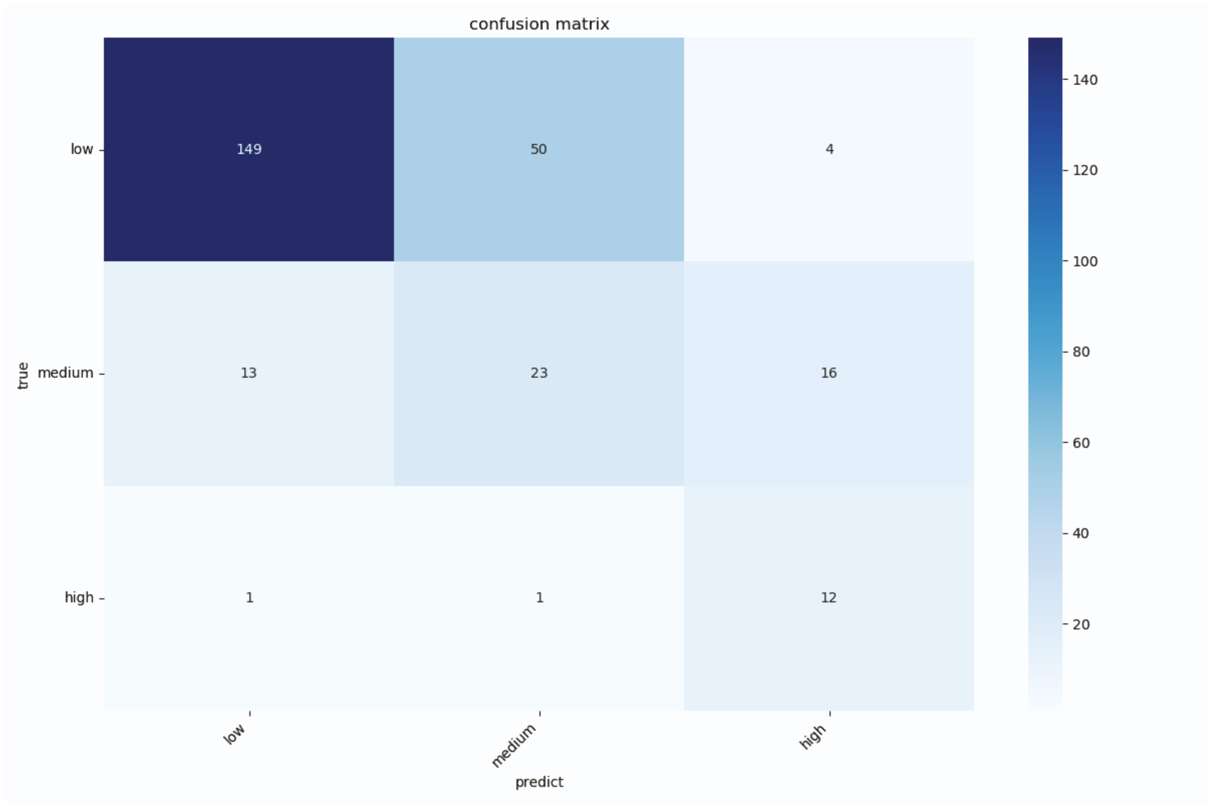

Fig. S5 Confusion matrix of eXtreme Gradient Boosting(XGBoost) for normalized tick report frequency prediction based on K-Means clustering method.

Supplementary Tables

Table S1: List of covariates used for tick report frequency and Tick-Borne Encephalitis(TBE) modeling.

covariates (source)		description
Climate covariates (ERA5-Land daily aggregated climate reanalysis dataset)	soil temperature (warm season) (°C)	Temperature of the soil in layer 1 (0 - 7 cm)
	soil temperature (cold season) (°C)	
	precipitation (warm season) (mm)	Accumulated liquid and frozen water, including rain and snow, that falls to the Earth's surface.
	precipitation (cold season) (mm)	
	humidity (warm season) (%)	the amount of water vapor present in the air
	humidity (cold season) (%)	
	DTR (warm season) (°C)	Diurnal Temperature Range
	DTR (cold season) (°C)	
Environmental covariates (MOD13Q1.061 and European	NDVI (warm season)	Normalized Difference Vegetation Index

Environment Agency)	NDVI (cold season)	
	NHR	Normalized Habitat Richness
Socioeconomic covariates (Sweden statistics database)	dog registration information	registered dog numbers in each family
	rural population	population live in rural area
	recreation land use (km^2)	areas for leisure, sports
	total land use (km^2)	proportion or intensity of human-modified land types
	summer house	vacation home
	Gini coefficient	a numerical measure of wealth or income inequality
	citizen science tick data	human-reported tick observations

Table S2. Covariates selection by accuracy (Gaussian Mixture Model (GMM) categories) for Generalized Linear Model(GLM). Covariates are included (+) or excluded (-) sequentially (from left to right), according to the model selection criterion outlined in the method section, to the base set of covariates (in supplemental information Table S15) for the tick report frequency modeling. The final set of covariates for the selected GLM model includes: Base covariate set +humidity warm+DTR warm+precipitation cold+total land use+precipitation warm. Max tick report frequency in 2023 are used for training and max tick report frequency in 2024 are used for testing as offset. Note: DTR: Diurnal Temperature Range.

	baseline model	+humidity warm season	+DTR warm season
Accuracy	37.170%	64.310%	70.260%

Note: The model trained on NDVI in the warm season, soil temperature in the warm season and rural population is the baseline model.

	+precipitation cold	+total land use	+precipitation warm season
Accuracy	70.630%	71.00%	71.380%

Table S3. Covariates selection by accuracy (Gaussian Mixture Model (GMM) categories) for Generalized Additive Model(GAM). Covariates are included (+) or excluded (-) sequentially (from left to right), according to the model selection criterion outlined in the method section, to the base set of covariates (in supplemental information Table S15) for the tick report frequency modeling. The final set of covariates for the selected GAM model includes: Base covariate set+ precipitation warm+ soil temperature warm.

	baseline model	+precipitation warm	+soil temperature warm
Accuracy	56.130%	62.450%	62.830%

Note: The model trained on NDVI in the warm season, soil temperature in the warm season and rural population is the baseline model.

Table S4. Covariates selection by accuracy (Gaussian Mixture Model (GMM) categories) for Least Absolute Shrinkage and Selection Operator(LASSO) regression. Covariates are included (+) or excluded (-) sequentially (from left to right), according to the model selection criterion outlined in the method section, to the base set of covariates (in supplemental information Table S15) for the tick report frequency modeling. The final set of covariates for the selected LASSO regression model includes: Base covariate set+ soil temperature cold.

	baseline model	+soil temperature cold
Accuracy	62.450%	63.570%

Note: The model trained on NDVI in the warm season, soil temperature in the warm season and rural population is the baseline model.

Table S5. Covariates selection by accuracy (Gaussian Mixture Model (GMM) categories) for spatial Least Absolute Shrinkage and Selection Operator(LASSO) regression. Covariates are included (+) or excluded (-) sequentially (from left to right), according to the model selection criterion outlined in the method section, to the base set of covariates (in supplemental information Table S15) for the tick report frequency modeling. The final set of covariates for the selected spatial LASSO regression model includes: Base covariate set+ soil temperature cold+ summer house+humidity cold+ Gini coefficient +DTR summer. Note: DTR: Diurnal Temperature Range; NDVI:Normalized Difference Vegetation Index .

	baseline model	+soil temperature cold	+summer house
Accuracy	62.450%	64.310%	65.060%

Note: The model trained on NDVI in the warm season, soil temperature in the warm season and rural population is the baseline model.

	+humidity cold	+ Gini coefficient	+DTR summer
Accuracy	65.430%	65.800%	66.910%

Table S6. Covariates selection by accuracy (Gaussian Mixture Model (GMM) categories) for decision tree. Covariates are included (+) or excluded (-) sequentially (from left to right), according to the model selection criterion outlined in the method section, to the base set of covariates (in supplemental information Table S15) for the tick report frequency modeling. The final set of covariates for the selected decision tree model includes: Base covariate set+ dog registration. Note: NDVI:Normalized Difference Vegetation Index.

	baseline model	+dog registration
Accuracy	35.090%	67.290%

Note: The model trained on NDVI in the warm season, soil temperature in the warm season and rural population is baseline model.

Table S7: Covariates selection by accuracy (Gaussian Mixture Model (GMM) categories) for XGBoost. Covariates are included (+) or excluded (-) sequentially (from left to right), according to the model selection criterion outlined in the method section, to the base set of covariates (supplemental information Table S15) for the tick report frequency modeling. The final set of covariates for the selected eXtreme Gradient Boosting(XGBoost) model includes: Base covariate set+summer house+dog information+NHR. Note: NHR: Normalized Habitat Richness.

	baseline model	+summer house	+dog information	+NHR
Accuracy	56.510%	65.430%	66.910%	68.770%

Table S8. Hyperparameters of each model in tick report frequency modeling. Note: LASSO: Least Absolute Shrinkage and Selection Operator; XGBoost: eXtreme Gradient Boosting

LASSO regression	L1 term	0.1
Spatial LASSO	spatial error term	1
Decision tree	layer depth	3
XGBoost	gamma	0.3
	learning rate	0.1
	max depth	9
	min child weight	1.2
	n estimators	10
	subsample	0.5

Table S9. Hyperparameters of each model in Tick-Borne Encephalitis(TBE) cases modeling. Note: LASSO: Least Absolute Shrinkage and Selection Operator; XGBoost: eXtreme Gradient Boosting

LASSO regression	L1 term	0.1
Spatial LASSO	spatial error term	1

Decision tree	layer depth	4
XGBoost	gamma	0.5
	learning rate	0.1
	max depth	7
	min child weight	1
	n estimators	10
	subsample	0.7

Table S10. Covariate selection by accuracy (Gaussian Mixture Model (GMM) categories) for Generalized Linear Model(GLM) on the TBE dataset. Covariates are included (+) or excluded (-) sequentially (from left to right), according to the model selection criterion outlined in the method section, to the base set of covariates (supplemental information Table S16). The final set of covariates for the selected GLM model includes: Base covariate set+recreation land use.

	baseline model	+ recreation land use
Accuracy	60.220%	61.340%

Table S11. Covariate selection by accuracy (Gaussian Mixture Model (GMM) categories) for Generalized Additive Model(GAM) on the TBE dataset. Covariates are included (+) or excluded (-) sequentially (from left to right), according to the model selection criterion outlined in the method section, to the base set of covariates (supplemental information Table S16). The final set of covariates for the selected GAM model includes: Base covariate set+DTR warm+NDVI warm. Note: DTR: Diurnal Temperature Range; NDVI: Normalized Difference Vegetation Index.

	baseline model	+ DTR warm	+NDVI warm
Accuracy	63.200%	65.430%	68.030%

Table S12. Covariate selection by accuracy (Gaussian Mixture Model (GMM) categories) for Least Absolute Shrinkage and Selection Operator(LASSO) regression on the Tick-Borne Encephalitis(TBE) dataset. Covariates are included (+) or excluded (-) sequentially (from left to right), according to the model selection criterion outlined in the method section, to the base set of covariates (supplemental information Table S16). The final set of covariates for the selected LASSO regression model includes: Base covariate set+ recreation land use+ summer house+ precipitation warm+ humidity cold+ Gini coefficient.

	baseline model	+ recreation land use	+summer house	+precipitation warm	+humidity cold	+Gini coefficient

Accuracy	57.990%	59.850%	60.590%	62.080%	62.830%	63.94%

Table S13. Covariates selection by accuracy (Gaussian Mixture Model (GMM) categories) for spatial Least Absolute Shrinkage and Selection Operator(LASSO) regression on the Tick-Borne Encephalitis(TBE) dataset. Covariates are included (+) or excluded (-) sequentially (from left to right), according to the model selection criterion outlined in the method section, to the base set of covariates (supplemental information Table S16). The final set of covariates for the selected spatial LASSO regression model includes: Base covariate set+ summer house+ precipitation warm+ recreation land use+ humidity cold+ Gini coefficient.

	baseline model	+ summer house	+precipitation warm	+recreation land use	+humidity cold	+Gini coefficient
Accuracy	58.360%	60.220%	60.970%	61.710%	62.450%	63.94%

Table S14. Covariate selection by accuracy (Gaussian Mixture Model (GMM) categories) for eXtreme Gradient Boosting(XGBoost) on the Tick-Borne Encephalitis(TBE) dataset. Covariates are included (+) or excluded (-) sequentially (from left to right), according to the model selection criterion outlined in the method section, to the base set of covariates (supplemental information Table S16). The final set of covariates for the selected XGBoost model includes: Base covariate set+precipitation warm+total land use.

	Base covariate set	+ precipitation warm	+total land use	-tick report frequency
Accuracy	62.450%	72.120%	73.610%	70.260%

Table S15 Generalized Linear Model (GLM) regression tick report frequency model results. Note: DTR: Diurnal Temperature Range; NDVI: Normalized Difference Vegetation Index; NHR: Normalized Habitat Richness. Asterisks indicate statistically significant coefficients (*p < 0.05) based on a two-sided Wald z-test, as computed in the negative binomial GLM (statsmodels, Python).

	coefficient	std err	z	P>|z|	[0.025	0.975]
soil temperature warm	4.3841	1.698	2.582	**0.010**	1.057	7.711
soil temperature cold	-0.205	0.582	-0.352	0.724	-1.345	0.935
precipitation warm	-0.696	0.775	-0.898	0.369	-2.216	0.823
precipitation cold	0.635	1.034	0.615	0.539	-1.391	2.662
humidity warm	-1.164	4.667	-0.249	0.803	-10.312	7.983
humidity cold	-1.928	5.179	-0.372	0.710	-12.079	8.222
DTR warm	-1.280	1.611	-0.795	0.426	-4.438	1.876
DTR cold	-1.138	1.386	-0.821	0.412	-3.856	1.579

NDVI warm	3.339	1.133	2.948	**0.003**	1.119	5.560
NDVI cold	-1.146	0.755	-1.517	0.129	-2.627	0.335
dog registration information	2.696	1.400	1.926	0.054	-0.047	5.440
rural population	1.935	0.633	3.055	**0.002**	0.694	3.177
recreation land use	0.474	1.124	0.423	0.673	-1.728	2.677
total land use	1.497	0.980	1.527	0.127	-0.424	3.419
summer house	-1.567	0.909	-1.725	0.084	-3.349	0.213
NHR	0.664	1.292	0.514	0.607	-1.867	3.196
Gini coefficient	1.265	1.224	1.034	0.301	-1.133	3.664

Table S16 Generalized Linear Model (GLM) regression Tick-borne encephalitis (TBE) cases model results. Note: DTR: Diurnal Temperature Range; NDVI: Normalized Difference Vegetation Index; NHR: Normalized Habitat Richness. Asterisks indicate statistically significant coefficients (*p < 0.05) based on a two-sided Wald z-test, as computed in the negative binomial GLM (statsmodels, Python).

Covariates	coefficient	std err	z	P>|z|	[0.025	0.975]
soil temperature warm	9.928	3.643	2.726	**0.006**	2.789	17.068
soil temperature cold	-3.452	0.906	-3.810	**0.000**	-5.229	-1.676
precipitation warm	1.680	1.192	1.409	0.159	-0.657	4.017
precipitation cold	-1.926	1.704	-1.131	0.258	-5.266	1.413
humidity warm	-2.698	7.080	-0.381	0.703	-16.575	11.178
humidity cold	-4.285	7.574	-0.566	0.572	-19.129	10.559
DTR warm	-0.398	2.510	-0.159	0.874	-5.318	4.522
DTR cold	-7.998	2.655	-3.012	**0.003**	-13.203	-2.794
NDVI warm	0.049	1.784	0.028	0.978	-3.448	3.547
NDVI cold	3.503	1.141	3.072	**0.002**	1.268	5.739
dog registration information	4.063	1.730	2.349	**0.019**	0.673	7.454
rural population	2.374	0.830	2.860	**0.004**	0.747	4.002
recreation land use	-1.125	1.493	-0.754	0.451	-4.052	1.801
total land use	-0.827	2.263	-0.366	0.715	-5.263	3.608

summer house	1.1935	1.065	1.121	0.262	-0.894	3.281
NHR	1.903	2.225	0.856	0.392	-2.457	6.265
Gini coefficient	-0.177	1.731	-0.103	0.918	-3.571	3.216
tick report frequency	2.838	1.234	2.300	**0.021**	0.419	5.256

Table S17. Model comparison on the binary categories tick reports 2024 testset. Note: GLM: Generalized Linear Model; GAM: Generalized Additive Model; XGBoost: eXtreme Gradient Boosting; LASSO: Least Absolute Shrinkage and Selection Operator.

	baseline model (GLM)	GLM	GAM	LASSO regression	spatial LASSO regression	decision tree	XGBoost
Accuracy	86.160%	92.190 %	91.820 %	88.480%	88.480%	82.160%	75.840%

Table S18. Model comparison of GLM and GAM on the 3-class categories tick reports 2024 test set. Note: GLM: Generalized Linear Model; GAM: Generalized Additive Model

	GLM* (selected model)	GAM*
Accuracy	71.380%	62.830%

Note: * represents max tick report frequency in 2023 is used for training and max tick report frequency in 2024 is used for testing as offset.

Methods S1. Lasso regression spatial error model

Let $X = \{x_1, x_2, ..., x_n\}$ represents the set of covariate values for each of the n municipalities and each $x_i \in \mathbb{R}^{1\times m}$ is a vector containing the covariates values of the municipality i. Let $y \in \mathbb{R}^{n\times 1}$ denote the vector containing the tick report frequency in each municipality. Given the coefficient $\beta \in \mathbb{R}^{m\times 1}$ for linear regression, the error of a municipality is $\varepsilon_i = \left|y_i - x_i\beta\right|$. The error set E can be represented as $\mathrm{E} = \{\varepsilon_1, \varepsilon_2, ..., \varepsilon_n\}$. Thus the error can be formulated as

$$J_e = \frac{1}{2}\sum_{j,k=1}^{N} w_{j,k}(\varepsilon_j - \varepsilon_k)^2 \quad (1)$$

where, $w_{j,k}$ is the connection between municipality j and municipality k. If j and k are neighbours, weight $w_{j,k}$ is 1, otherwise it is 0. Therefore, the whole loss function of the lasso regression can be represented as follows:

$$\begin{aligned} J_{lasso} &= \|y - X\beta\|^2 + \lambda_1|\beta| + \lambda_2 J_e \quad (2) \\ &= \|y - X\beta\|^2 + \lambda_1|\beta| + \lambda_2 \frac{1}{2}\sum_{j,k=1}^{N} w_{j,k}(\varepsilon_j - \varepsilon_k)^2 \\ &= \|y - X\beta\|^2 + \lambda_1|\beta| + \lambda_2(\sum_{j=1}^{N} \varepsilon_j^2 w_{j,j} - \sum_{j,k=1,j\neq k}^{N} \varepsilon_j\varepsilon_k w_{j,k}) \\ &= \|y - X\beta\|^2 + \lambda_1|\beta| + \lambda_2(\mathrm{E}^T D\mathrm{E} - \mathrm{E}^T W\mathrm{E}) \\ &= \|y - X\beta\|^2 + \lambda_1|\beta| + \lambda_2\mathrm{E}^T L\mathrm{E} \end{aligned}$$

where, $L = D - W$ is called the Laplacian graph. λ_1 is the coefficient to balance L1 regularization term of LASSO and the MSE loss. λ_2 is the coefficient to balance the graph regularization term and the MSE loss.

The loss function J_{lasso} can be optimized by gradient descent[1].

$$\frac{dJ_{lasso}(\beta)}{d\beta} = 2X^T|y - X\beta| + \lambda_1 sign(\beta) + 2\lambda_2 X^T L(y - X\beta) \quad (3)$$

1. Bengio, Y., Lamblin, P., Popovici, D. & Larochelle, H. Greedy layer-wise training of deep networks. *Adv. Neural Inf. Process. Syst.* **19**, (2006).
